# Epigenetic regulation of starvation-induced autophagy in *Drosophila* by histone methyltransferase G9a

**DOI:** 10.1038/s41598-017-07566-1

**Published:** 2017-08-04

**Authors:** Phan Nguyen Thuy An, Kouhei Shimaji, Ryo Tanaka, Hideki Yoshida, Hiroshi Kimura, Eiichiro Fukusaki, Masamitsu Yamaguchi

**Affiliations:** 1 Department of Biotechnology, Graduate School of Engineering, Osaka University, 2-1 Yamadaoka, Suita, Osaka 565-0871 Japan; 2 Department of Applied Biology, Kyoto Institute of Technology, Kyoto, 606-8585 Japan; 3 The Center for Advanced Insect Research Promotion, Kyoto Institute of Technology, Kyoto, 606-8585 Japan; 4 Departmetnt of Biological Sciences, Graduate School of Bioscience and Biotechnology, Tokyo Institute of Technology, Nagatsuta, Midori-ku, Yokohama 226-8501 Japan

## Abstract

Epigenetics is now emerging as a key regulation in response to various stresses. We herein identified the *Drosophila* histone methyltransferase G9a (dG9a) as a key factor to acquire tolerance to starvation stress. The depletion of dG9a led to high sensitivity to starvation stress in adult flies, while its overexpression induced starvation stress resistance. The catalytic domain of dG9a was not required for starvation stress resistance. dG9a plays no apparent role in tolerance to other stresses including heat and oxidative stresses. Metabolomic approaches were applied to investigate global changes in the metabolome due to the loss of dG9a during starvation stress. The results obtained indicated that dG9a plays an important role in maintaining energy reservoirs including amino acid, trehalose, glycogen, and triacylglycerol levels during starvation. Further investigations on the underlying mechanisms showed that the depletion of dG9a repressed starvation-induced autophagy by controlling the expression level of Atg8a, a critical gene for the progression of autophagy, in a different manner to that in cancer cells. These results indicate a positive role for dG9a in starvation-induced autophagy.

## Introduction

Appropriate responses to stressful conditions are crucial for the adaptation of an organism to environmental challenges. Epigenetic regulation in response to these conditions has been attracting increasing attention due to its rapid and long-lasting effects on gene expression in response to environmental changes without alterations in DNA sequences^1^. In a wide variety of organisms, epigenetic regulation has been found to mediate long-term effects on gene expression or the transgenerational inheritance of phenotypic changes caused by various stresses^2^. In terms of starvation stress, prenatal starvation was initially reported to alter DNA methylation marks on the imprinted gene *IGF2* and this change persisted throughout the human life-span^3^. Other studies also suggested that *Sirtuin 1* (*Sirt1*), a histone deacetylase, plays a role in responses to starvation stress in *Drosophila* ^4^. However, except for *Sirt1*, the epigenetic factors that are critical in responses to starvation stress have not yet been identified. The histone methyltransferase G9a was recently reported to mediate autophagy by regulating the expression of autophagy-related genes including *LC3B*, *WIPI*, and *DOR* in starved human pancreatic cancer cells^5^. Since autophagy is an intracellular degradation system that responds to starvation, the signaling pathways mediating starvation-induced autophagy have been elucidated in detail^6^. However, gene regulation of the components in these signaling pathways has not been fully examined *in vivo*.

G9a was initially reported in 2001 as a mammalian histone lysine methyltransferase accounting for methylation at lysine 9 (K9) of histone H3 (H3K9) *in vitro* ^7^. In the last decade, the epigenetic regulation of gene expression via post-translational modifications by G9a in diverse biological processes has been a rapidly growing research subject. G9a may specifically associate with euchromatin and catalyze the mono-, di-, and trimethylation of H3K9, which is involved in transcriptional gene silencing^8–11^. This function of G9a was found to be important for early embryogenesis in mice^12–15^, the propagation of imprints^16^, and control of DNA methylation^17, 18^. Moreover, G9a is functional in the survival of mammalian cells under hypoxic stress^19^. These findings demonstrate the importance of G9a during development as well as in stress tolerance in mammals. However, the embryonic lethal phenotype has made it difficult to study the function of mammalian G9a at later developmental stages. Thus, the role of G9a on longevity under stress conditions in living organisms currently remains unknown.

In *Drosophila melanogaster*, G9a (dG9a) has been shown to play an important role in neural development as well as behavioral processes such as learning and memory^20^. A previous study using a *dG9a* null mutant strain (*dG9a* ^*RG*5^) demonstrated that dG9a was not essential for *Drosophila* viability or fertility^21^; however, embryogenesis was delayed^22^. It is important to note that while there are various environmental stresses in the wild, *Drosophila* is always maintained under optimal conditions in the laboratory. Therefore, dG9a may play a critical role in environmental stress tolerance. On the other hand, a relationship has been proposed between gene regulation, nutrition, and metabolism because many enzymes involved in epigenetic gene regulation require co-substrates generated by cellular metabolism^23, 24^. Similarly, environmentally-induced epigenetic responses may induce changes in metabolism in an organism in order to support adaptation or stress tolerance^25, 26^. Hence, metabolomics may be used as a fundamental method to assess changes in the metabolic pathways of an organism and provide an insight into the dynamics of cellular functions that contribute to the survival of an organism in nature^27^.

In the present study, we exposed flies lacking dG9a to various stress conditions. We found that dG9a-depleted flies were specifically sensitive to starvation, but not heat or oxidative stress. In order to explain these results, the global metabolic profiling of fasted wild-type and dG9a-depleted flies was performed to elucidate the metabolic changes that occur when dG9a is removed.

The main changes in cellular metabolites accounting for energy generation under starvation stress were observed in dG9a-depleted flies. Further investigations showed that the loss of dG9a repressed starvation-induced autophagy by controlling the expression level of Atg8a in a methyltransferase-independent manner. This regulation by dG9a appears to be opposite to that reported previously in mammalian pancreatic cancer cells in which human G9a negatively regulated autophagic cell death^5^.

## Materials and Methods

### Fly stocks

All fly stocks were reared at 25 °C on standard food (0.7% agar, 10% glucose, 4% dry yeast, 5% cornmeal, 3% rice bran). Canton S was used as the wild-type. *dG9a* ^*RG5*^ and *dG9a* ^*del34*^ flies were kindly provided by Dr. P. Spierer and Dr. C. Seum. *dG9a* ^*RG5*^ flies were backcrossed 10 times with Canton S to adjust the genetic background to Canton S. A mutant allele was recovered with the adjacent *w* ^*1118*^ marker. We confirmed that the*w* ^*1118*^ marker did not reduce fly viability, life span, or survival (data not shown). The *UAS-FLAG-IR dG9a* (strain #79) fly stock was produced previously^28^. The *FB-GAL4* (*y w; GAL4* ^*fat*^) strain was kindly provided by Ronald P. Kühnlein^29^. We generated a fly strain carrying *GAL80* ^*ts*^ and *FB-GAL4* (*y w; P(GAL4)fat; GAL80* ^*ts.αTub84B*^) by crossing. The *Atg8a* ^*d4*^ strain is kindly provided by Gabor Juhasz^30^. The *Atg8a* ^*d4*^ mutant lacks the first 25 codons of Atg8a^30^. All other stocks used in this study were obtained from the Drosophila Genetic Resource Center in Kyoto and Bloomington Drosophila Stock Center.

### Starvation assay

In the starvation assay, 3–5-day-old adult flies were placed into vials including a piece of paper soaked in 1.0 mL PBS and the number of living flies was monitored until all had died. All assays were performed under non-crowded conditions (<20 flies per vial) and the results from several independent assays were combined. The median survival time was calculated and graphs were generated with the Kaplan-Meier method by GraphPad Prism 6 software. Significance was calculated with Log-rank tests using GraphPad Prism 6 software. In rescue assays with glucose, glucose was dissolved in PBS in vials.

In the starvation assay utilizing temperature-sensitive Gal80, flies were grown at 18 °C on normal food as described previously^31^. Three-five-day-old adult male flies were placed into vials including a piece of paper soaked in 1.0 mL PBS in a temperature controlled incubator at 29 °C and the number of living flies was monitored until all had died.

### Stress assays

In the oxidative stress assay, newly eclosed adult flies were transferred on instant food (Carolina) with 10 mM paraquat (Nacalai Tesque). Every 3 days, they were transferred to new vials containing fresh instant food and the number of living flies was monitored until all had died. In the heat stress assay, 3–5-day-old adult flies in vials containing fresh instant food were placed into a temperature controlled incubator at 36 °C and the number of living flies was monitored until all had died. All assays were performed under non-crowded conditions (20 flies per vial).

### GC-MS and LC-MS analyses

Samples were collected after 0, 12, 24, and 36 h of fasting. Samples were washed, freeze-dried, and kept at −80 °C until analyzed. A 5-mg dry weight of each sample was homogenized using a ball mill (5 min, 20 Hz). There were 4–5 biological replicates for each condition. One milliliter of extraction solvent (2.5 methanol: 1 ultra-pure water: 1 chloroform) was used for each sample to extract a wide range of low molecular hydrophilic metabolites. After centrifugation (16,000 x *g* for 3 min), 900 µL supernatant was transferred to a 1.5-mL micro tube and mixed with 400 µL ultra-pure water (Wako). Centrifugation was performed again to separate the polar and non-polar phases. The polar phase was transferred into two fresh 1.5-mL Eppendorf tubes (400 µL/tube) for GC-MS and LC-MS analyses.

In the GC-MS analysis, samples were spun using centrifugal concentration (VCe36S, Taitec Co., Tokyo, Japan) for 2 h in order to remove the solvent and then freeze-dried for 16 h. Samples underwent derivatization by methoxyamine hydrochloride (Sigma Aldrich, St. Louis, MO, USA) in pyridine (50 µL, 10 mg/mL) at 30 °C for 90 min, and then by 50 µL of N-methyl- trimethylsilyltrifluoroacetamide (MSTFA) (GL Sciences, Tokyo, Japan) at 37 °C for 30 min. The GC-MS analysis was performed using the same conditions described in our previous study on *Drosophila* embryos^32^.

In the LC-MS analysis, samples were concentrated into 50 µL by centrifugal concentration and analyzed within 24 h of extraction. The analysis was performed using the Shimadzu Nexera UHPLC system coupled with LCMS 8030 Plus (Shimadzu, Kyoto, Japan) using the conditions described in our previous study^33^.

### Data analysis for metabolic profiling

A principle component analysis (PCA), Partial Least Square projection to the latent structure discriminant analysis (PLS-DA), and orthogonal projections to latent structures discriminant analysis (OPLS-DA) were performed by utilizing SIMCA-P^+^ version 13 (Umetrics, Umea, Sweden). Unit variance was used as the scaling method and no transformation was performed. The good quality of the OPLS-DA model was designated by goodness-of-fit (*R* ^*2*^) and goodness-of-prediction (*Q* ^*2*^) values greater than 0.9.

The PLS-DA model was considered to be valid after permutations when the *R* ^*2*^ *Y*-intercepts and *Q* ^*2*^-intercepts did not exceed 0.3–0.4 and 0.05, respectively^34^.

The OPLS-DA model was then validated using CV-ANOVA (Analysis of variance testing of cross-validated residuals) and two groups of samples were significantly different when *p*(CV-ANOVA) < 0.05. Important metabolites from this model were defined based on the combination of VIP (Variable Importance in the Projection) score and p(corr) (loading scaled as a correlation coefficient between the model and original data). The criteria for the cut-off point were VIP > 1.0 and |p(corr)| > 0.5^35^.

An independent two-way ANOVA test was used to assess whether the candidates obtained from OPLS-DA were significantly different. The two-way ANOVA test was performed using the “Time series Analysis” function of Metabolyst 3.0^36^, a web-based metabolomic data processing tool (available for free at http://www.metaboanalyst.ca).

A Hierarchical Cluster Analysis (HCA) was conducted using Multiexperiment View Version 4.9^37^ (Dana-Farber Cancer Institute, Boston, MA, USA, available for free at the website http://www.tm4.org/mev.html). In this study, similarities between two objects were obtained without losing generality based on the Euclidean distance.

### Measurement of glycogen and triacylglycerol (TAG)

Glycogen and TAG levels were measured as described previously^38–41^. In the glycogen assay, 5 flies were homogenized in 200 μL of a solution containing 2% sodium sulfate and 800 μL of a chloroform/methanol (1:1) mixture was added to the homogenate. After centrifugation at 6,000 x *g* for 3 min, the pellet was dissolved in 1 mL of anthrone reagent (1.4 g of anthrone in 1 L of 72% H_2_SO_4_). The solution was heated at 100 °C for 17 min and analyzed in a spectrometer at 625 nm. In the TAG assay, 10 adult flies were homogenized in 200 μL of a solution containing 0.1% Tween 20 and the homogenate was heated at 70 °C for 5 min. After centrifugation at 15,000 x *g* for 5 min, 200 μl of TAG reagent (Sigma) was added to 50 μL of the supernatant and incubated at 37 °C for 30 min. The incubated solution was analyzed in a spectrophotometer at 540 nm. The remaining homogenate was used for protein quantification by the Bradford assay with bovine serum albumin as a standard. The amount of TAG was normalized to the protein amount.

### Immunostaining analysis

Three to five adult flies were dissected in PBS and fixed for 20 min in 4% paraformaldehyde at 25 °C. After permeabilization with 0.3% Triton X-100 in PBS, samples were incubated with primary antibodies specific for Atg8a (1:200, Novus Biologicals), dG9a (1:500, produced previously^40^), or H3K9me2 (1/400, previously produced and used for *Drosophila* cells^22, 42^) at 4 °C for 16 h. After washing with 0.3% Triton X-100 in PBS, samples were incubated with the secondary antibody, Alexa Fluor 594 anti-mouse IgG (1:400, Molecular Probes) at 25 °C for 2 h. Samples were mounted with VECTASHIELD mounting media with DAPI (Vector Laboratories, Inc.). Intensity was only measured from nuclei in interphase and with a z-axis clearly present in their centers. These nuclei were selected with DAPI signals. We measured the intensities of signals from 10 nuclei derived from at least 3 adult flies. Preparations were examined using confocal microscopy (Olympus FV10i). The images obtained were analyzed with MetaMorph (Molecular Devices).

### Flip-out clonal analysis

We generated mosaic gene expression in larval fat body with flip-out clone generation system^43^. *hsFLP; Act5C* > *DC2* > *GAL4, UAS-GFP* flies were crossed with *Atg8a* RNAi strain *Atg8a* ^*HMS01328*^. 0–36 h embryos were collected and heat-shocked for 30 min at 37 °C at 36 h after egg laying. Third instar larva were put into a dish containing a piece of paper and 1 mL PBS for 6 h and immunostained as described above after dissection.

### Western immunoblot analysis

Fifteen adult flies were homogenized in 100 μL of 2x SDS sample buffer (2% SDS, 10% glycerol, 0.002% BPB, and 0.063 M Tris-HCl), heated at 95 °C for 2 min, and centrifuged at 15,000 x g at 4 °C for 10 min. Five microliters of supernatants was fractionated by 15% SDS-PAGE and transferred to PVDF membranes (Biorad). The membranes were blocked with 0.3% dry milk in Tris-buffered saline containing Tween 20 (TBS-T) (137 mM NaCl, 2.7 mM KCl, 25 mM Tris-HCl, and 0.1% Tween 20, pH 7.6) and incubated with an anti-Atg8a antibody (1:1000, Cell Signaling) or anti-α-tubulin antibody (1:10000, DSHB) at 4 °C for 16 h. Membranes were washed with TBS-T and incubated with HRP-conjugated anti-rabbit or mouse IgGs (Thermo Scientific) for 60 min. After washing membranes with TBS-T, visualization was performed with ECL-select (GE Healthcare). Signal intensity measurements were performed with a CS analyzer (ATTO).

### Reverse transcription-quantitative PCR (RT-qPCR)

RNAs were isolated using Trizol^®^ reagent (Invitrogen) from adult whole bodies. cDNA was synthesized using the PrimeScript RT reagent kit (TaKaRa) according to the manufacturer’s instructions. qPCR was performed with SYBR^®^ Premix Ex Taq^TM^ II (TaKaRa) using CFX96 touch^TM^ (Biorad), and data were analyzed with the ΔΔCt method. *β-tubulin* was used as an internal control. The primer sequences of the genes examined are listed below.

*dG9a* forward 5′-TCAGATGGCCTATCTCCTTC-3′ (used in Fig. S1B)

*dG9a* reverse 5′-CAGTCCGCAGTTCATAATCC-3′ (used in Fig. S1B)

*dG9a* forward 5′-ACCGATGACAGCTACTACTTTG-3′ (used in Fig. 2F)

*dG9a* reverse 5′-TGTAGTCCTGATGCTCGTAGA-3′ (used in Fig. 2F)

*β-tubulin* forward 5′-ATACGGTGACCTGAACCATC-3′

*β-tubulin* reverse 5′-TACTCGGACACCAGATCG-3′

*CG6262* forward 5′-GGATCGGGTACACAGCTATTC-3′

*CG6262* reverse 5′-CGGGAACGAGAAGGTGAAA-3′

*G6P* forward 5′-CTGGGAAGTTACCTGGGAATTAG-3′

*G6P* reverse 5′-AAACAGCTGAGACCGCATAG-3′

*HEXA* forward 5′-GGATCGGGTACACAGCTATTC-3′

*HEXA* reverse 5′-CGGGAACGAGAAGGTGAAA-3′

*Pgi* forward 5′-TCGAGAAGAATGCTCCTGTTATC-3′

*Pgi* reverse 5′-GCAAGTACTGATCGTAGGGAAG-3′

*fbp* forward 5′-GCCGGAGAAGGGAAAGATATAC-3′

*fbp* reverse 5′-TCTTGGCCGCAATGTAGTT-3′

*Gapdh1* forward 5′-ATGTCTCCGTTGTGGATCTTAC-3′

*Gapdh1* reverse 5′-CCTCGACCTTAGCCTTGATTT-3′

*Pgk* forward 5′-GCTGAACAAGGAGCTGAAGTA-3′

*Pgk* reverse 5′-TCTCAATCAGCTGGATCTTGTC-3′

*Atg8a* forward 5′-TACCAGGAACATCACGAGGA-3′

*Atg8a* reverse 5′-CGACCGGAGCAAAGTTAGTTA-3′

## Results

### dG9a^RG5^ null mutant flies are sensitive to starvation

**Figure 1:**
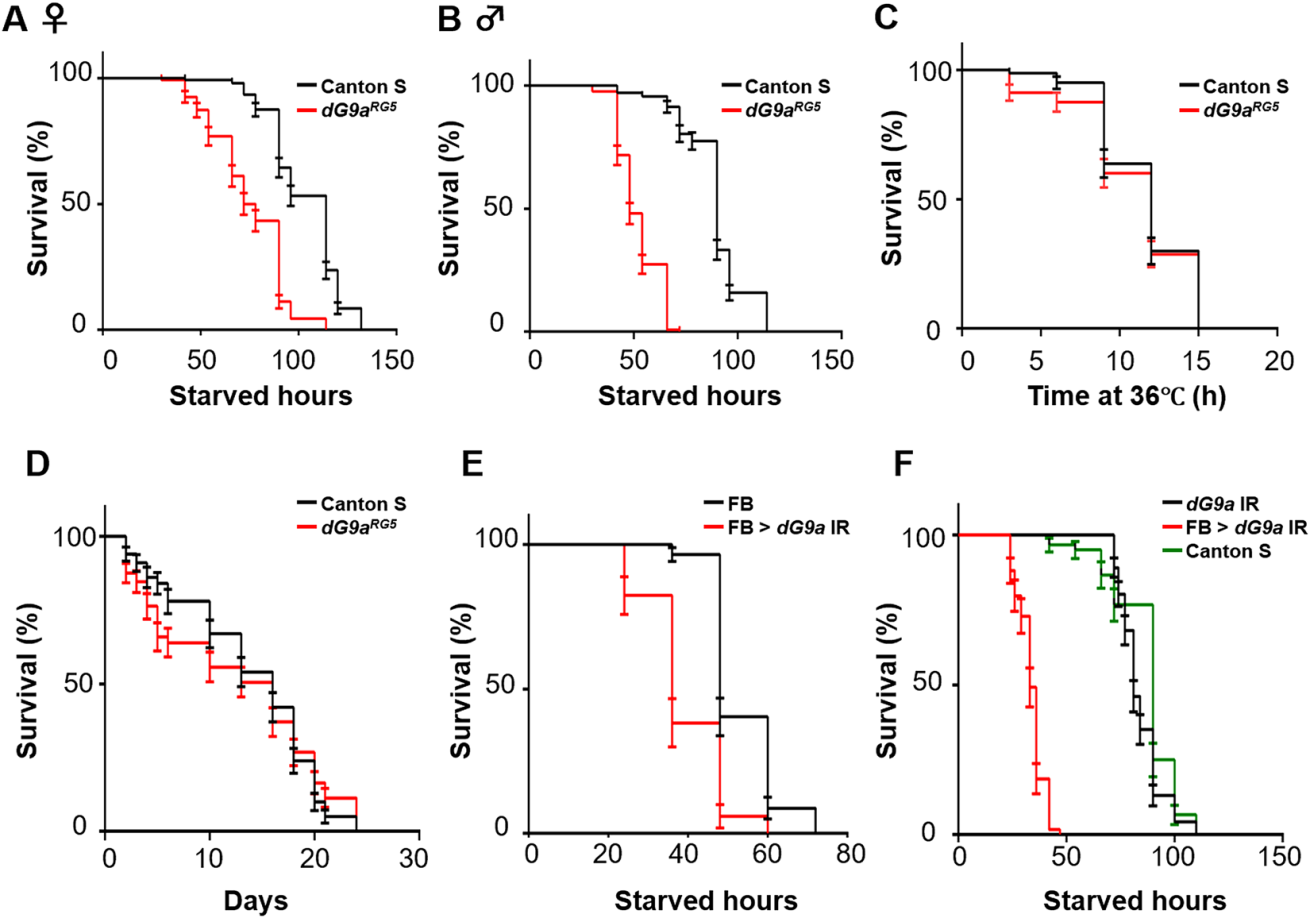
dG9a is critical for survival under starvation stress. (**A**) The results of a viability assay under starvation conditions using females of the wild-type (Canton S) (n = 153 from 8 independent experiments) and dG9a null mutant (dG9a^RG5^) (n = 135 from 7 independent experiments) P < 0.0001 (**B**). The results of a viability assay under starvation conditions using males of the wild-type (n = 131 from 7 independent experiments) and dG9a null mutant (dG9a^RG5^) (n = 138 from 7 independent experiments) P < 0.0001. (**C**) The results of a viability assay under heat stress using males of the wild-type (n = 80 from 4 independent experiments) and dG9a null mutant (dG9a^RG5^) (n = 80 from 4 independent experiments) P = 0.41. (**D**) The results of a viability assay under oxidative stress using males of the wild-type (n = 100 from 5 independent experiments) and dG9a null mutant (dG9a^RG5^) (n = 97 from 5 independent experiments) P = 0.80. (**E**) The results of a viability assay under starvation conditions using males of the FB (+; FB-GAL4; +) (n = 57 from 3 independent experiments) and FB > dG9a IR (w; UAS-dG9a IR/FB-GAL4; +) (n = 34 from 2 independent experiments) P < 0.0001. (**F**) The results of a viability assay under starvation conditions using males of the FB > dG9a IR (w; UAS-dG9a IR/FB-GAL4; +) (n = 59 from 3 independent experiments), males of the dG9a IR (w; UAS-dG9a IR/+; +) (n = 92 from 5 independent experiments) and males of Canton S (n = 60 from 3 independent experiments). dG9a IR and Canton S: P > 0.05. (**A**–**F**) Error bars represent standard errors (SE).

Previous studies using the dG9a null mutant strain (*dG9a* ^*RG*5^) showed that dG9a was not essential for *Drosophila* viability or fertility^21^; however, *dG9a* has been shown to regulate the expression of a wide variety of genes *in vivo*. In order to investigate epigenetic regulation by dG9a in response to various environmental changes, the adult wild-type (Canton S) and *dG9a* ^*RG5*^ null mutant strain were examined under several challenging environmental conditions including heat exposure, oxidative stress, and starvation. We found that *dG9a* ^*RG5*^ mutant flies were exclusively sensitive to fasting conditions, but not heat or oxidative stress (Fig. 1A–D). The median survival times of *dG9a* ^*RG5*^ mutant males (48 h) and females (78 h) were 47% and 32% shorter, respectively, than those of wild-type males (90 h) and females (114 h) under starvation stress (Fig. 1A,B). However, the viability of the larval *dG9a* ^*RG5*^ mutant under starvation stress was not significantly less than that of the wild-type (Fig. S1A). Under heat shock or oxidative stress conditions, the viability of the *dG9a* ^*RG5*^ mutant was not significantly different from that of the wild-type (Fig. 1C,D). These results indicate that dG9a plays an important role in the survival of adult flies during starvation stress conditions.

The fat body functions as a major store in response to nutrition demand in insects^44^. In order to clarify whether the function of *dG9a* in the fat body is critical for fly viability under starvation stress, we performed a viability assay utilizing fat body-specific *dG9a* knockdown flies generated with the GAL4-UAS targeted expression system^45^. The efficient knockdown of *dG9a* was confirmed by RT-qPCR (Fig. S1B). We also confirmed that the fat body-specific driver (FB-GAL4) properly induced the expression of the target protein in the adult fat body by immunostaining with the anti-GFP antibody utilizing the strain carrying the FB-GAL4 and UAS-GFP constructs (Fig. S1C). The median survival time of fat body-specific *dG9a* knockdown flies (FB > *dG9a IR* in Fig. 1E, median survival time: 36 h) was 25% shorter than that of flies that have only FB-GAL4 driver (FB in Fig. 1E, median survival time: 48 h). The median survival time of *FB-GAL4/*+ flies (48 h) is much shorter than that of Canton S (90 h). The generation of GAL4 protein induces rapid energy consumption, which may result in the decrease of starvation stress tolerance in *FB-GAL4/*+ flies. Furthermore, *FB-GAL4* transgene is inserted in the genomic region of *Pyruvate carboxylase* (*PCB*) gene. The insertion may result in the decrease of starvation stress tolerance in *FB-GAL4/*+ flies, since PCB is required for gluconeogenesis and lipogenesis. We also performed starvation assay utilizing the fat body-specific *dG9a* knockdown flies (FB > *dG9a IR* in Fig. 1F), flies that have only *UAS-dG9a IR* transgene (*dG9a IR* in Fig. 1F) and wild type flies. The median survival time of *dG9a-IR* flies was not significantly different from that of Canton S (Fig. 1F), therefore we concluded that the mutations of *dG9a-IR* flies do not affect the survival time. These results indicate that the function of dG9a in the fat body is critical for fly viability under starvation stress. These results also confirmed that the decrease observed in the viability of the *dG9a* ^*RG5*^ mutant under starvation stress, shown in Fig. 1A,B, was not due to a possible background mutation in the *dG9a* ^*RG5*^ mutant. We further confirmed that the trans-heterozygous combination of *dG9a* ^*RG5*^ and *dG9a* ^*del34*^ alleles (*dG9a* ^*RG5*^/*dG9a* ^*del34*^) shows severe reduction of starvation tolerance compared to wild type under fasting conditions (Fig. S1D). The data support the conclusion that the reduction of starvation tolerance of *dG9a* ^*RG5*^ is not due to the background mutations of *dG9a* ^*RG5*^ flies. However, the rescue of the *dG9a* ^*RG5*^ mutant by a genomic DNA fragment containing the whole *dG9a* gene may be necessary to further confirm this conclusion.

**Figure 2:**
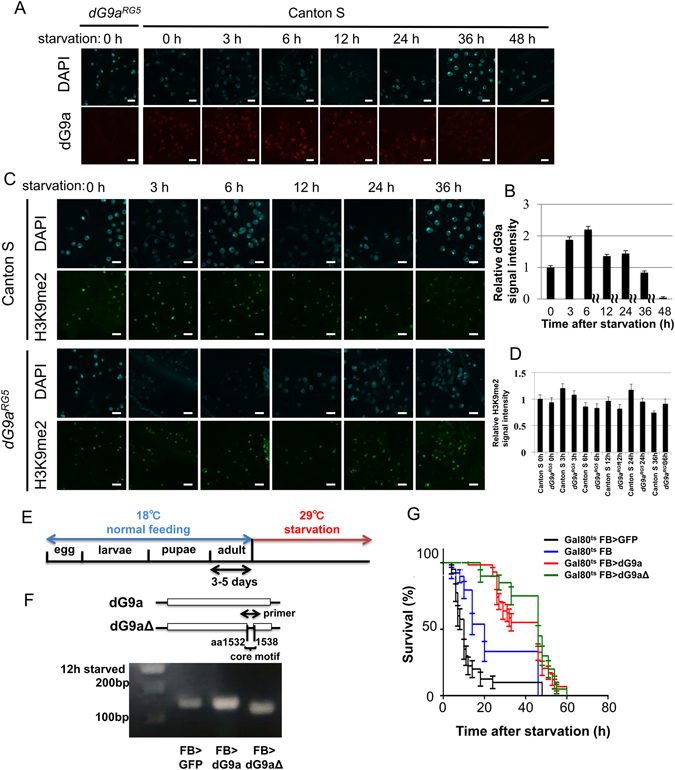
dG9a function in the fat body is critical for adult fly viability under starvation stress. (**A**) Transient induction of dG9a in the adult fat body under starvation conditions. Fat bodies of the wild-type and dG9a^RG5^ mutant were immunostained with an anti-dG9a antibody (red) and stained with DAPI (cyan). The dG9a^RG5^ mutant was used as a negative control. (**B**) Quantification of dG9a signals in nuclei of the wild-type fat body. Values were adjusted by subtracting background fluorescence n = 10. (**C**) Quantification of H3K9me2 signals in nuclei of the wild-type and dG9a^RG5^ mutant fat bodies. Values were adjusted by subtracting background fluorescence (n = 10). (**D**) H3K9me2 signal in the adult fat body under starvation conditions. Fat bodies of the wild-type and dG9a^RG5^ mutant were immunostained with an anti-H3K9me2 antibody (green) and stained with DAPI (cyan). (**E**) The experimental design of dG9a overexpression under starvation conditions. (**F**) Confirmation of dG9a and dG9a_Δ1532–1538_ overexpression by semi-quantitative RT-PCR after 12 h of starvation. The primer included the core motif of the SET domain of dG9a. dG9a was more strongly expressed in FB > dG9a (FB-GAL4/GAL80^ts.αTub84B^; UAS-dG9a/+) than in FB > GFP (a control: FB-GAL4/GAL80^ts.αTub84B^; UAS-GFP/+). The amplicon size is 142 bp in FB > dG9a and FB > GFP. The amplicon size of FB > dG9aΔ(FB-GAL4/GAL80^ts.αTub84B^; UAS-dG9a_Δ1532–1538_/+) is 21 bp shorter because of the lack of the core motif of the SET domain. The full-length gel image is shown in Fig. S6A. (**G**) The results of a viability assay under starvation conditions using males of the control (GAL80^ts^ FB > GFP: FB-GAL4/+; GAL80^ts.αTub84B^/UAS-GFP) (n = 40 from 2 independent experiments), another control (GAL80^ts^ FB: FB-GAL4/+; GAL80^ts.αTub84B^/+) (n = 39 from 2 independent experiments), a dG9a overexpression line (GAL80^ts^ FB > dG9a: FB-GAL4/+; GAL80^ts.αTub84B^/UAS-dG9a) (n = 58 from 3 independent experiments), and dG9aΔ_1532–1538_ overexpression line (GAL80^ts^ FB > dG9aΔ: FB-GAL4/+; GAL80^ts.αTub84B^/UAS-dG9a_Δ1532–1538_) (n = 40 from 2 independent experiments). Significant differences were observed between GAL80^ts^ FB > GFP and GAL80^ts^ FB > dG9a (P < 0.0001) and between GAL80^ts^ FB > GFP and GAL80^ts^ FB > dG9aΔ (P < 0.0001). Significant differences were also noted between GAL80^ts^ FB and GAL80^ts^ FB > dG9a (P < 0.0001) and between GAL80^ts^ FB and GAL80^ts^ FB > dG9aΔ (P < 0.0001). No significant differences were found between GAL80^ts^ FB > dG9a and GAL80^ts^ FB > dG9aΔ (P = 0.23). (**B,C,G**) Error bars represent SE.

In order to reveal the localization of dG9a in the fat bodies of starved adult flies, we immunostained fat bodies with the anti-dG9a antibody (Fig. 2A). dG9a signals were detected in nuclei and signal intensity increased up to 6 h after starvation (Fig. 2B). These results indicate that dG9a localizes in the nuclei of fat body cells and may play a role in gene expression under starvation stress. However, the overall intensity of H3K9me2 in the nuclei of fat body cells under starvation was not significantly affected by the loss of dG9a (Fig. 2C,D). These results suggest that dG9a regulates gene expression in fat body nuclei in a H3K9 methyltransferase activity-independent manner. However, we cannot exclude the possibility that the absence of a significant difference in H3K9me2 levels was due to the insufficient detection thresholds of immunostaining analyses. More sensitive analyses, such as a chromatin immunoprecipitation sequencing analysis, will be required in order to investigate this possibility.

In an attempt to clarify whether the overexpression of dG9a extends survival times under starved conditions, we performed a viability assay utilizing a temporal control system of dG9a overexpression by temperature-sensitive GAL80^31^. dG9a or GFP, as a control, was only overexpressed after the initiation of starvation (Fig. 2E). In order to examine whether the methyltransferase activity of dG9a is critical, we also overexpressed dG9a_Δ1532–1538_ lacking the catalytic core motif of the SET domain, which is necessary for the histone methyltransferase activity of dG9a^28^. The overexpression of *dG9a* and *dG9a*_*Δ1532–1538*_ was confirmed by semi-quantitative RT-PCR (Fig. 2F). The overexpression of dG9a or dG9a_Δ1532–1538_ extended survival times under starvation stress over those of the control strains (GAL80^ts^ FB > GFP and GAL80^ts^ FB strains in Fig. 2G) (P < 0.001), and no significant difference was observed in the viabilities of the *dG9a* and *dG9a*_*Δ1532–1538*_ overexpression lines (*P* = 0.23) (Fig. 2G). The shorter survival times of FB and FB > GFP strains (Fig. 2G) than that of the wild-type Canton S (Fig. 1) may have been due to the higher temperature used for the GAL80/GAL4 system since the temperature directly affects the central metabolism of *Drosophila* ^46^. Another possibility could be the production of additional proteins such as GAL80, GAL4, and GFP. We also performed same starvation assays utilizing flies that carry only UAS construct. No significant difference in survival time was observed among these flies (Fig. S1E), therefore we concluded that the mutations of these flies did not affect the survival time. In any event, these results suggest that the catalytic activity of dG9a is not required for the acquisition of starvation stress resistance by dG9a.

### Differences between wild-type and dG9a^RG5^ mutant flies under starvation could be explained by the composition of the metabolome

In order to obtain a general perspective on changes caused by the loss of dG9a, we performed metabolic profiling of fasted wild-type and *dG9a* ^*RG5*^ mutant by employing a set of targeted and non-targeted analytical methods using IP-LC-MS/MS and GC-Q/MS, respectively. Since male flies of the *dG9a* ^*RG5*^ mutant were more sensitive to starvation stress (Fig. 1A,B), only unmated male flies were used and samples were collected after 0, 12, 24, and 36 h of fasting. With our platforms, 83 low-molecular-weight hydrophilic metabolites belonging to central metabolic pathways were detected (Fig. 2A).

**Figure 3:**
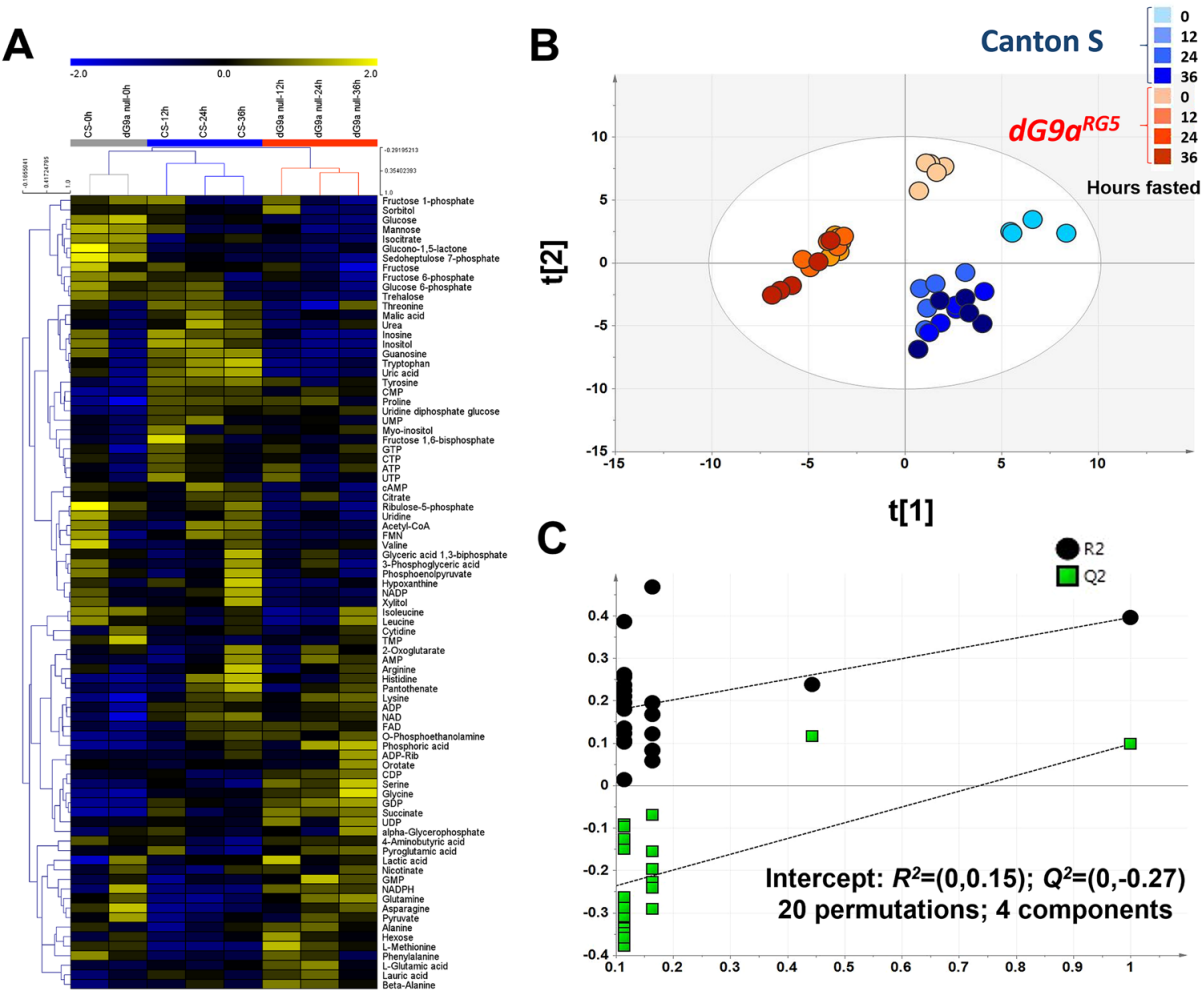
Fasted dG9a^RG5^ mutant flies show a distinct metabolic profile. (**A**) HCA data showing changes in the cellular metabolites of wild-type and dG9a^RG5^ mutant flies under starvation; the color scale is plotted on the top of the figure. Before starvation, flies with both genotypes shared similar profiles and were grouped into one cluster (grey). During starvation, flies with each genotype showed different profiles and were discriminated hierarchically into two clusters, wild-type (blue) and dG9a^RG5^ mutant (red). A similar discrimination based on genotypes was observed on the score plot of the supervised analysis PLS-DA (**B**). In each genotype, samples collected before fasting were clearly separated from samples collected during fasting. (**C**) Statistical validation by the permutation test with 20 permutations of PLS-DA (R^2^Y-intercepts = (0,0.15); Q^2^-interceps = (0, −0.27)).

HCA was constructed to provide a global view of the metabolic state of fasted control and dG9a null mutant flies (Fig. 3A). Due to the horizontal axis of the HCA results, it was clear that the two strains shared similar metabolic profiles before starvation (at 0 h); therefore, they were grouped into the same grey cluster. However, during starvation, the expression patterns of metabolites varied between the two strains, resulting in the separation of Canton S and *dG9a* ^*RG5*^ into two different clusters, blue and orange, respectively. A similar discrimination was again observed in the supervised discriminant analysis PLS-DA (Fig. 3B). In the PLS-DA model with the eight different groups (wild-type and *dG9a* ^*RG5*^ mutant at four different time points), we found that samples were mainly discriminated into two groups based on genotypes. Nevertheless, within each group, samples at 0 h, representing samples not exposed to starvation, were completely separated from those after 12, 24 and 36 h of fasting. PLS-DA model was considered valid after permutation since the *R* ^*2*^ *Y*-intercepts and *Q* ^*2*^-interceps did not exceed 0.3–0.4 and 0.05, respectively (Fig. 3C).

Collectively, these results indicated that prior to starvation, both strains shared similar metabolic profiles. However, the loss of dG9a caused the altered cellular metabolic profile in *Drosophila* during starvation.

### The loss of dG9a causes major changes in amino acid metabolism

**Figure 4:**
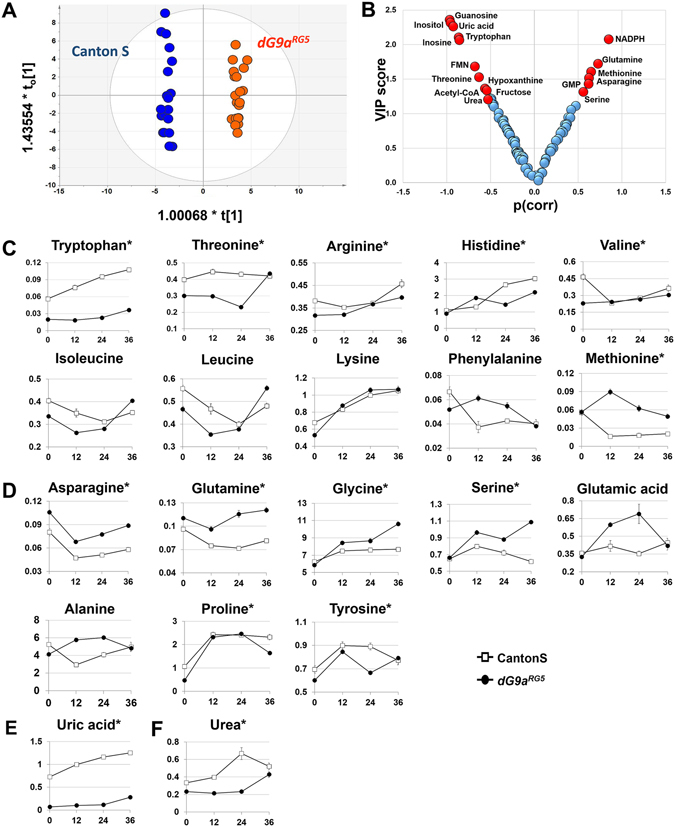
Amino acid metabolism. A general view of the metabolites detected belonging to amino acids including: (**A**) The OPLS-DA score plot showed a clear separation between two genotypes with p(CV-ANOVA) = 1.99925E-23. (**B**) A plot formed by VIP scores and p(corr) revealed using OPLS-DA. The criteria VIP > 1.0 and |p(corr)|>0.5 were used to select potential metabolites (red) showing marked changes due to the loss of dG9a. (**C**) Essential amino acids (**D**) Non-essential amino acids (**E**) Uric acid (**F**) Urea. 4–5 replications for each time point. *The metabolites were significantly different in “genotype” between the wild-type and dG9a^RG5^ mutant in a two-way ANOVA with P < 0.05.

In an attempt to identify the metabolites affected by the loss of function of dG9a, OPLS-DA was generated to maximize differences between wild-type and *dG9a* ^*RG5*^ mutant flies. This model was constructed with one predictive and two orthogonal components, which showed a clear separation between the groups along the predictive components (Fig. 4A). In this model, important metabolites related to the function of dG9a under fasting conditions were proposed (Fig. 4B; Table S1), and were found to be related to amino acid metabolism.

Similar to mammals, 20 amino acids found in the proteins of *Drosophila* may also be classified into essential and non-essential amino acids^47^. We herein demonstrated that the levels of many essential amino acids in dG9a-depleted flies were lower than in those in the wild-type during 36 h of fasting (Fig. 4C). Significant differences were observed in the expression levels of tryptophan, threonine, arginine, histidine, and valine between the fasted wild-type and *dG9a* ^*RG5*^ mutant with the *p-value* for the genotype being less than 0.05 in the two-way ANOVA. Since there was no nutrient supplement during fasting, the only source of free essential amino acids was from protein degradation. The levels of non-essential amino acids do not reflect the actual rate of proteolysis because they may be synthesized or derived from essential amino acids. Moreover, the expression levels of uric acid and urea, the waste products of amino acid and nitrogen metabolism, increased overtime and were significantly higher in the wild-type than in the *dG9a* ^*RG5*^ mutant (Fig. 4E,F). Thus, these results suggested that in contrast to the wild-type strain, dG9a-depleted flies were unable to maintain an appropriate protein degradation rate, which is an essential process for recycling amino acids for translation or energy generation under fasting. On the other hand, asparagine, glutamine, and methionine levels were significantly higher in dG9a-depleted flies (Fig. 4C,D). These amino acids were reported to be inhibitors of the autophagic pathway in studies using isolated rat hepatocytes^48^. A high level of L-glutamine was previously reported to be important for the activation of the target of rapamycin (TOR) pathway, which suppresses autophagy^49^.

Collectively, these results suggest that the loss of dG9a affects the ability to recycle amino acids via protein degradation in starvation-induced autophagic pathways, which may be the reason why the viability of the mutants was lower than that of the wild-type strain under starvation stress.

### dG9a plays an important role in maintaining energy reservoirs during starvation

**Figure 5:**
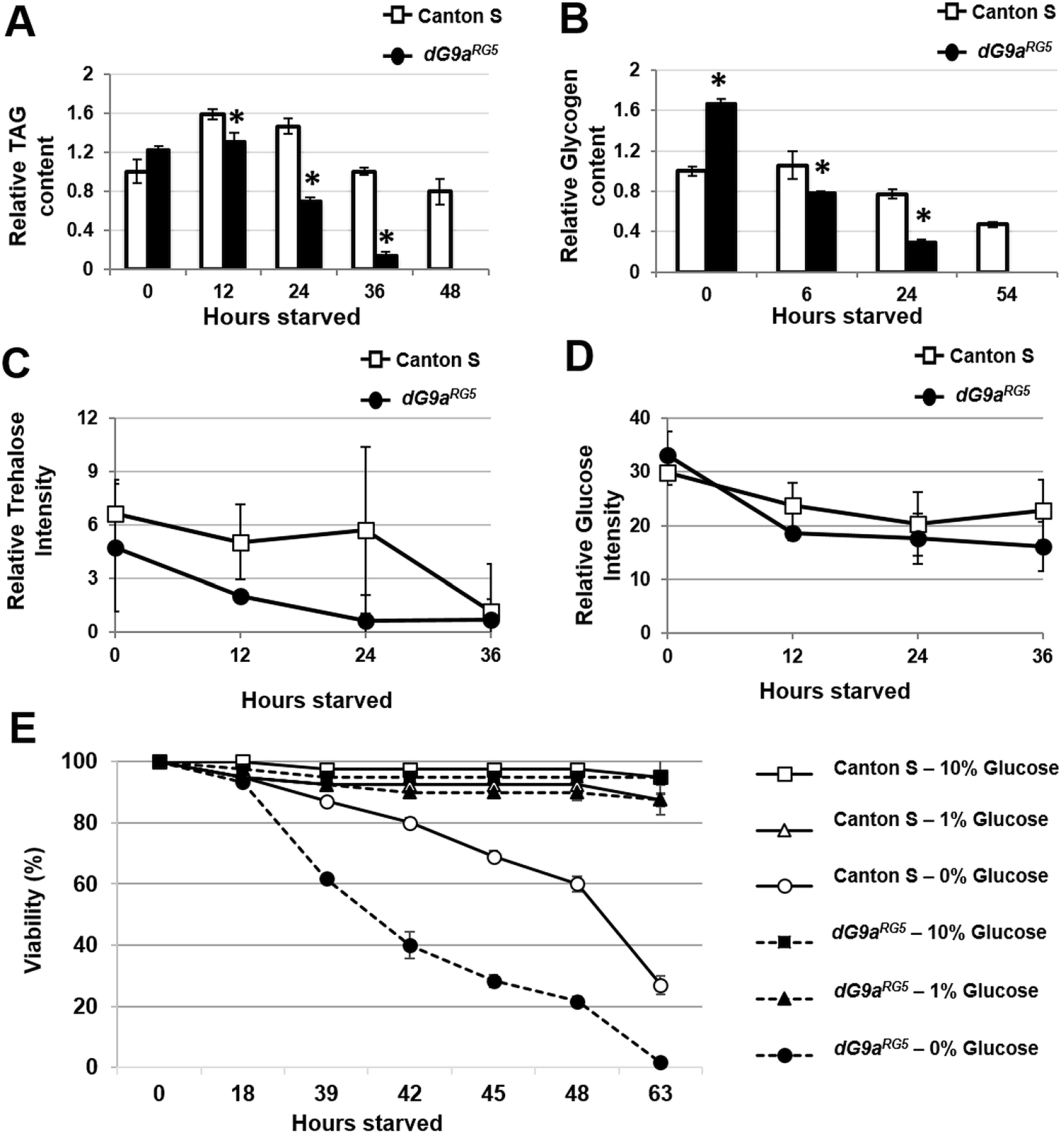
The loss of dG9a affects energy homeostasis in Drosophila during fasting. (**A**) Relative levels of TAG in the wild-type and dG9a^RG5^ mutant under starvation stress n = 3. *P < 0.05. (**B**) Relative levels of glycogen in the wild-type and dG9a^RG5^ mutant under starvation stress n = 5. *P < 0.05. The levels of trehalose (**C**) and glucose (**D**) from the GC-MS analysis. Only the level of trehalose showed a significantly different “genotype” between the wild-type and dG9a^RG5^ mutant in a two-way ANOVA with P = 9.17E-03. (**E**) The results of a viability assay with glucose supply (n = 40). When dG9a^RG5^ mutant flies were fed 1% and 10% glucose in PBS, they recovered their sensitivity and had similar viability to the wild-type.

The responses of cells to nutrient deprivation via autophagy involve a non-selective process of bulk cytoplasmic degradation^50, 51^. Hence, if our hypothesis is correct, these changes will also appear in metabolites related to lipid and carbohydrate sources. In *Drosophila*, TAG and glycogen are the main primary forms of energy sources stored in the fat body, analogous to the mammalian liver and white adipose tissues. In response to the energy needs of the body, trehalose will be synthesized in the fat body, excreted into the hemolymph, and utilized to produce glucose^52^. As predicted, when we measured TAG and glycogen levels, we found that they were lower in dG9a-depleted than in wild-type flies after 12 h of fasting (Fig. 5A,B). Metabolic profiling results revealed that trehalose levels were also significantly lower in the *dG9a* ^*RG5*^ mutant with a *p-value* = 0.01 for the genotype in the two-way ANOVA throughout starvation stress (0–36 h) (Fig. 5C). If these variations were caused by the mechanisms used to consume the reservoirs, the level of ATP may differ between the two strains. However, no significant difference was detected in the levels of glucose, the initial material for glycolysis, the major energy currency molecule of cells produced from glycolysis and the TCA cycle (Fig. 5D). When the levels of metabolites together with the mRNA levels of enzymes related to glycolysis were measured, no significant difference was observed between wild-type and dG9a-depleted flies (Fig. S3). Collectively, these results suggested that the loss of dG9a did not affect the ability to use resources to generate energy via glycolysis, whereas it may have affected the capability to conserve energy sources, which quickly drains the body’s energy and leads to weak tolerance under starvation stress.

We then investigated whether glucose supplements have the ability to rescue the viability of dG9a-depleted flies. After hatching, adult flies of the wild-type and *dG9a* ^*RG5*^ mutant were maintained on standard food for 48 h and then shifted to either PBS or 1% and 10% glucose in PBS. As we predicted, the addition of glucose completely rescued the sensitivity of dG9a-depleted flies to starvation stress; they showed similar viability to the wild-type (Fig. 5E). These results confirmed that dG9a plays an important role in maintaining not only amino acid catabolism, but also overall energy reservoirs, which may be used to generate glucose for the body during starvation.

### dG9a controls starvation-induced autophagy by regulating the expression of Atg8a

**Figure 6:**
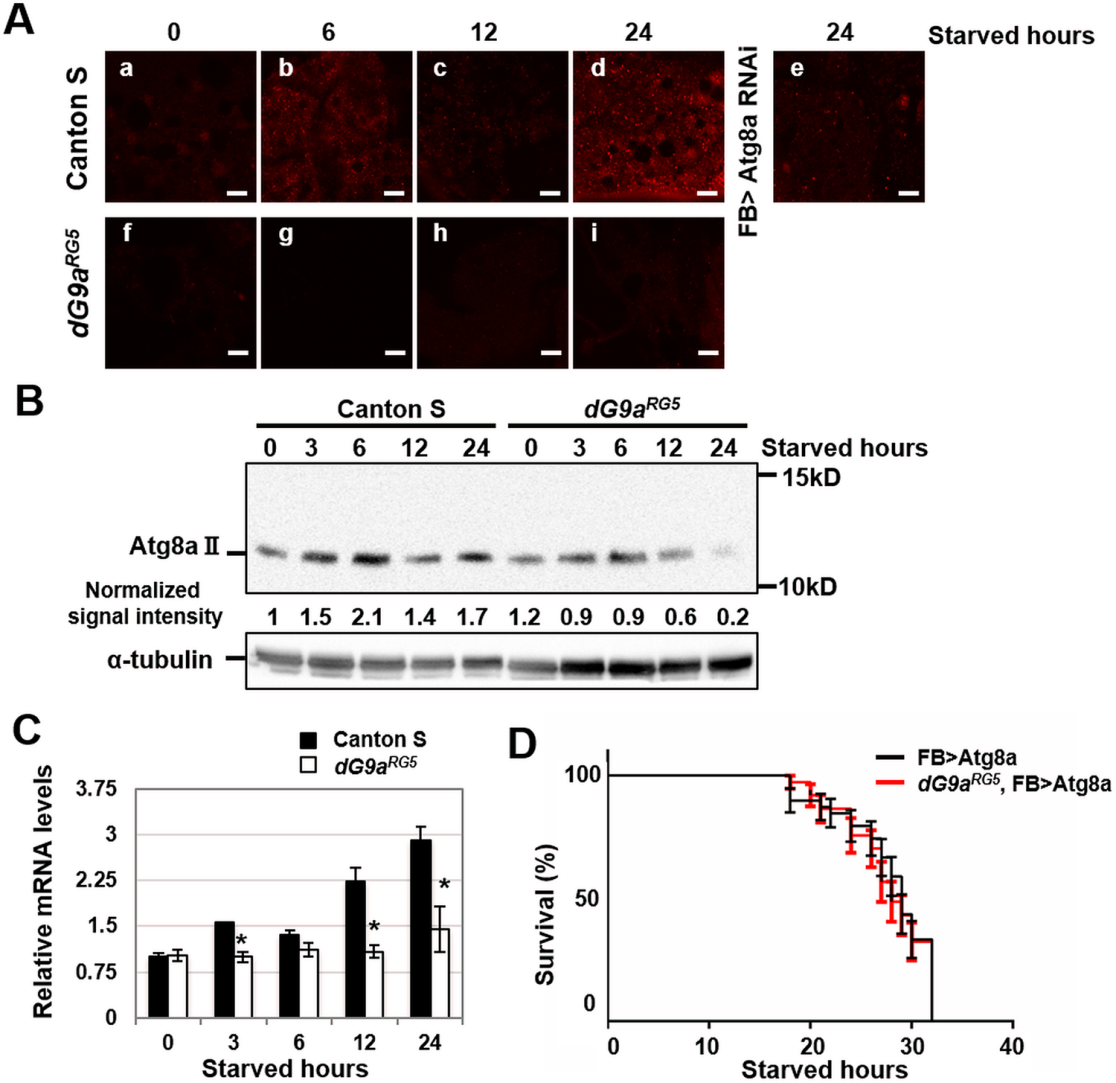
dG9a is responsible for the induction of autophagy under starvation stress. (**A**) Immunostaining of wild-type and dG9a^RG5^ mutant fat bodies under starvation conditions with an anti-Atg8a antibody. Strains: (a–d) Canton S (e) w; FB-GAL4/+; Atg8a^HMS01328^/+ (f–i) dG9a^RG5^. Starved hours: (a,f) 0 h (b,g) 6 h (c,h) 12 h (d,i) 24 h. Scale bars, 10 μm. (**B**) A western blot analysis of extracts from the starved wild-type and dG9a^RG5^ mutant. Blots were probed with anti-Atg8a and anti-α-tubulin antibodies. Signal intensity normalized with that of α-tubulin is shown. The full-length image of the blot is shown in Fig. S6B. (**C**) Quantification of mRNA levels by a RT-qPCR analysis of Atg8a in the starved wild-type and dG9a^RG5^ mutant. Results were normalized to α-tubulin and displayed as relative values to that of the 0-h starved wild-type n = 3. *P < 0.05. (**D**) The results of a viability assay under starvation conditions using the males of dG9a^RG5^, FB > Atg8a (dG9a^RG5^; FB-GAL4/Atg8a^Scer/UAS.P/T.T:Avic/GFP-EGFP,T:Disc/RFP-mCherry^; +) (n = 39 from 2 independent experiments) and FB > Atg8a (+; FB-GAL4/Atg8a^Scer/UAS.P/T.T:Avic/GFP-EGFP,T:Disc/RFP-mCherry^; +) (n = 38 from 2 independent experiments) strains P = 0.79. (**C,D**) Error bars represent SE.

Previous studies utilizing a human pancreatic cancer cell line showed that G9a regulates the expression of some autophagy genes *in vitro* ^5^. Autophagy occurs in the fat body in *Drosophila* under starvation stress^53^. Moreover, our metabolomic results indicated that the loss of dG9a affected the ability to recycle amino acids via protein degradation in starvation-induced autophagic pathways. Therefore, we focused on autophagy activity in *dG9a* ^*RG5*^ mutant under starvation stress. We immunostained fat bodies with anti-Atg8a IgG, a widely used marker for detecting autophagy activity. The inactive form of Atg8a (Atg8a-I) may be processed into its active form (Atg8a-II), which accumulates to form a dotted signal at the phagophore, an initial membrane for autophagosome membrane formation, the assembly site^54^. The antibody used in this study was more sensitive to Atg8a-II^55^. In the wild-type, the number of dotted signals of Atg8a increased after 6, 12, and 24 h of fasting (Fig. 6Aa–d). These dotted signals decreased in the fat bodies of fat body-specific *Atg8a* knockdown flies, confirming the specificity of anti-Atg8a IgG (Fig. 6Ad, e).

We confirmed the specificity of the anti-Atg8a antibody by generating somatic clones marked by GFP that express Atg8a dsRNA in larval fat body. The number of dot signals was clearly decreased in the RNAi clone area (Fig. S4A), which support the Atg8a staining specificity. In the *dG9a* ^*RG5*^ mutant, the number of dotted signals of Atg8a was less than that in the wild-type under starvation conditions (Fig. 6Aa–d,f–i). A Western blot analysis is widely used to evaluate autophagy activity. Atg8a-II(12 kDa) may be easily distinguished from Atg8a-I (15 kDa) by SDS-PAGE^54^. The western blot analysis further confirmed that the protein level of Atg8a-II was lower in the *dG9a* ^*RG5*^ mutant than in the wild-type (Fig. 6B). The mRNA level of Atg8a was significantly lower after 3, 12, and 24 h of fasting in the *dG9a* ^*RG5*^ mutant than in the wild-type (Fig. 6C). The Atg8a-II band was not detected in the *Atg8a* mutant (*Atg8a* ^*d4*^) (Fig. S4B), which validate the antibody specificity in the Western blot analysis.

*Atg8a* ^*d4*^ was also used as a control for the phenotype that lack of *Atg8a*. As predicted, similar levels of TAG, total protein, trehalose and glucose were observed between *dG9a* ^*RG5*^ and *Atg8a* ^*d4*^ at 24 h after starvation (Fig. S4C–F). Of note, the total protein levels after 24 h starving of both *dG9a* ^*RG5*^ and *Atg8a* ^*d4*^ were not significantly different, which validated our hypothesis that the lack of *dG9a* affected the ability to recycle amino acids via protein degradation. These results suggested that a decrease in the expression level of Atg8a causes a shortage in Atg8a for the progression of autophagy under starvation conditions in the *dG9a* ^*RG5*^ mutant. The specific overexpression of Atg8a in the fat body rescued the defect in viability in *dG9a* ^*RG5*^ mutant under starvation conditions (Fig. 6D). Furthermore, the level of energy reservoirs were also recovered and similar to control strain (Fig. S5). Therefore, dG9a is responsible for the increase required in the expression of *Atg8a* for sufficient autophagy activity under starvation conditions.

## Discussion

Previous studies revealed that G9a is important for early embryogenesis and essential for viability in mice^12–15^. G9a is also highly conserved among various metazoans including *Drosophila*, frogs (*Xenopus tropicalis*), fish (*Danio rerio*, *Tetraodon nigroviridis*, and *Takifugu rubripes*), and mammals. In *Drosophila*, although G9a is not essential for viability, the results of the present study suggest that *G9a* is conserved from the fly to mammals because of its importance in starvation stress tolerance, to which organisms are often exposed in the wild. This is also the first indication that epigenetic regulator-like G9a plays an essential role in the acquisition of starvation tolerance.

In order to clarify the underlying mechanisms by which the *dG9a* null mutant is more susceptible to starvation stress, “bottom up” approaches have been used. Non-targeted GC-MS-based and targeted LC-MS/MS-based metabolic profiling was performed to investigate changes in the metabolome due to the loss of dG9a. The results obtained from metabolic profiles showed that dG9a played important roles in maintaining energy homeostasis, the key factor for nutrient stress tolerance. dG9a modulated energy reservoirs including amino acid, trehalose, glycogen, and TAG levels during starvation via the autophagic process. One of the unique features of the adult *dG9a* ^*RG5*^ mutant is its higher content of glycogen under non-starved normal conditions than that of the wild-type (Fig. 6B). A previous study reported that the deletion of *G9a* in mouse adipose tissues promotes adipogenesis and increases body weight^56^. These findings and the present results suggest that *dG9a* is also responsible for the suppression of adipogenesis, similar to mammalian G9a. Further analyses are needed in order to clarify this point.

The results of the present study also indicated that dG9a controlled starvation-induced autophagy by activating the expression of Atg8a; however, dG9a generally represses gene expression by dimethylating H3K9. Previous studies reported that histone and non-histone protein methylation by G9a either activated or inhibited gene expression^57–62^. We also found that the catalytic activity of dG9a was not required for the acquisition of starvation stress resistance by dG9a. This is consistent with the results of immunostaining showing that H3K9me2 levels in the nuclei of fat body cells under starvation were not significantly affected by the loss of dG9a. G9a has also been reported to activate gene expression as a molecular scaffold for the assembly of transcriptional co-activators, and the catalytic domain of G9a is not required for this function^63^. Further studies are needed in order to clarify the mechanisms by which dG9a regulates the expression of Atg8a.

Similar *Atg8a* mRNA levels were observed after 6 h of fasting between wild-type and *dG9a* ^*RG5*^ mutant flies (Fig. 6C); however, Atg8a immunostaining signals was weaker in the *dG9a* ^*RG5*^ mutant than in the wild-type (Fig. 6A). Therefore, the loss of dG9a may repress the expression of genes that control Atg8a protein stability. Further studies are needed in order to elucidate the underlying mechanisms. During the development of *Drosophila*, metamorphosis is also a process that flies use to tolerate starvation stress. Even though our study demonstrated that dG9a is important for starvation stress tolerance, the viability of the *dG9a* ^*RG5*^ mutant was not significantly less than that of the wild-type during the pupal stage^21, 22^. Together with our results showing that the viability of the *dG9a* ^*RG5*^ mutant at the larval stage was not affected by fasting conditions, the function of dG9a for starvation stress appears to be specific to the adult stage. Since programmed autophagy during the 3^rd^ instar larval and pupal stages is well-known to be regulated by ecdysone through the PI3K pathway^64^, starvation-induced autophagy by dG9a in the adult stage may be operated by other pathways.

G9a is suggested to play a positive role in the promotion of tumorigenesis in various human cancer cells such as prostate^65^, leukemia^60^, lung^66^, breast^67^, and aggressive ovarian carcinoma^68^. The inhibition of G9a activity in cancer cells significantly inhibited cell proliferation by triggering cell cycle arrest, inducing apoptosis, or activating autophagic cell death^5, 69–72^. The novel results obtained in the present study on the role of dG9a to acquire starvation tolerance may also make it possible to explain the positive role of G9a in the promotion of tumorigenesis. Cells inside a tumor mass are exposed to starvation conditions because nutrients are not fully supplied to these cells^73, 74^. In order to overcome starvation stress, autophagy is induced in these cells^73, 74^. Therefore, G9a may play a role in the acquisition of starvation tolerance in cells in the tumor mass. In the present study, we found that the loss of dG9a led to the inactivation of starvation-induced autophagy due to a decrease in Atg8a levels. In contrast, a previous study on cancer cells showed that the loss of G9a during starvation activated the transcription of LC3B (the Atg8a ortholog in mammals) and triggered autophagy^5^. Taken together, these results suggest that the epigenetic gene regulation of G9a depends on cell/tissue types.

## Electronic supplementary material

Supplemental information
