# Supplementary material for: Epigenetic regulation of starvation-induced autophagy in Drosophila by histone methyltransferase G9a
Source: Sci Rep. 2017 Aug 4;7:7343. doi: 10.1038/s41598-017-07566-1 (PMC5544687; doi:10.1038/s41598-017-07566-1)
Supplement: Supplementary file 1 — Supplemental information [file 41598_2017_7566_MOESM1_ESM.pdf]

***Supplemental information:***

# Epigenetic regulation of starvation-induced autophagy in *Drosophila* by histone methyltransferase G9a

**Phan Nguyen Thuy An[1,*], Kouhei Shimaji [2,3,*], Ryo Tanaka [2,3], Hideki Yoshida[2,3], Hiroshi Kimura[4], Eiichiro Fukusaki[1,**], Masamitsu Yamaguchi[2,3, **]**

[1] Department of Biotechnology, Graduate School of Engineering, Osaka University, 2-1 Yamadaoka, Suita, Osaka, 565-0871, Japan.

[2] Department of Applied Biology, [3] The Center for Advanced Insect Research Promotion, Kyoto Institute of Technology, Kyoto, 606-8585, Japan.

[4]Departmetnt of Biological Sciences, Graduate School of Bioscience and Biotechnology, Tokyo Institute of Technology, Nagatsuta, Midori-ku, Yokohama, 226-8501, Japan.

* These authors contributed equally to this work

**Corresponding author

MY: Tel.: +81 75 724 7781; Fax: +81 75 724 7799; E-mail: myamaguc@kit.ac.jp

EF: Tel./ Fax: +81-6-6879-7424; E-mail: fukusaki@bio.eng.osaka-u.ac.jp

**Table S1. Potential metabolites affected due to the loss of *dG9a* during starvation.**

| Compound | OPLS-DA | | *p-value* from Two-way ANOVA | | |
|---|---|---|---|---|---|
| | p(corr) | VIP | Starved time | Genotype | Interaction |
| **Threonine** | -0.63 | 1.53 | 7.15E-04 | 2.45E-07 | 5.40E-03 |
| **Inositol** | -0.96 | 2.32 | 1.59E-05 | 6.88E-23 | 7.05E-03 |
| **Serine** | 0.56 | 1.32 | 1.23E-03 | 7.14E-06 | 7.05E-03 |
| **Methionine** | 0.65 | 1.61 | 4.65E-02 | 1.02E-06 | 7.54E-03 |
| **Uric acid** | -0.92 | 2.26 | 9.71E-07 | 2.42E-20 | 3.73E-02 |
| **Tryptophan** | -0.86 | 2.11 | 5.85E-06 | 3.43E-15 | 4.02E-02 |
| **Lactic acid** | 0.42 | 1.06 | 1.21E-02 | 1.62E-03 | 4.35E-02 |
| **Acetyl CoA** | -0.54 | 1.34 | 1.51E-02 | 1.08E-04 | 0.09 |
| **Fructose** | -0.53 | 1.21 | 2.39E-06 | 7.14E-06 | 0.08 |
| **Guanosine** | -0.97 | 2.36 | 0.12 | 1.01E-20 | 0.20 |
| **Glutamine** | 0.73 | 1.72 | 4.48E-02 | 1.02E-06 | 0.21 |
| **FMN** | -0.68 | 1.68 | 0.35 | 4.75E-06 | 0.23 |
| **Urea** | -0.50 | 1.22 | 3.94E-02 | 1.62E-03 | 0.22 |
| **NADP** | -0.49 | 1.20 | 0.19 | 2.77E-03 | 0.32 |
| **Xylitol** | -0.47 | 1.14 | 0.15 | 5.22E-03 | 0.38 |
| **NADPH** | 0.85 | 2.08 | 0.06 | 1.01E-10 | 0.41 |
| **Ru5P** | -0.48 | 1.14 | 8.52E-07 | 1.59E-05 | 0.55 |
| **cAMP** | -0.41 | 1.01 | 0.13 | 1.03E-02 | 0.52 |
| **Hypoxanthine** | -0.56 | 1.36 | 1.52E-02 | 2.35E-04 | 0.56 |
| **Pyruvate** | 0.47 | 1.11 | 1.24E-02 | 5.23E-03 | 0.56 |
| **GMP** | 0.62 | 1.43 | 1.31E-02 | 7.98E-05 | 0.62 |
| **Asparagine** | 0.63 | 1.52 | 4.70E-06 | 1.14E-06 | 0.91 |
| **G6P** | -0.46 | 1.10 | 9.09E-03 | 2.77E-03 | 0.91 |
| **Inosine** | -0.86 | 2.07 | 1.57E-02 | 9.41E-11 | 0.92 |
| **Malic acid** | -0.49 | 1.22 | 0.18 | 5.35E-03 | 0.97 |

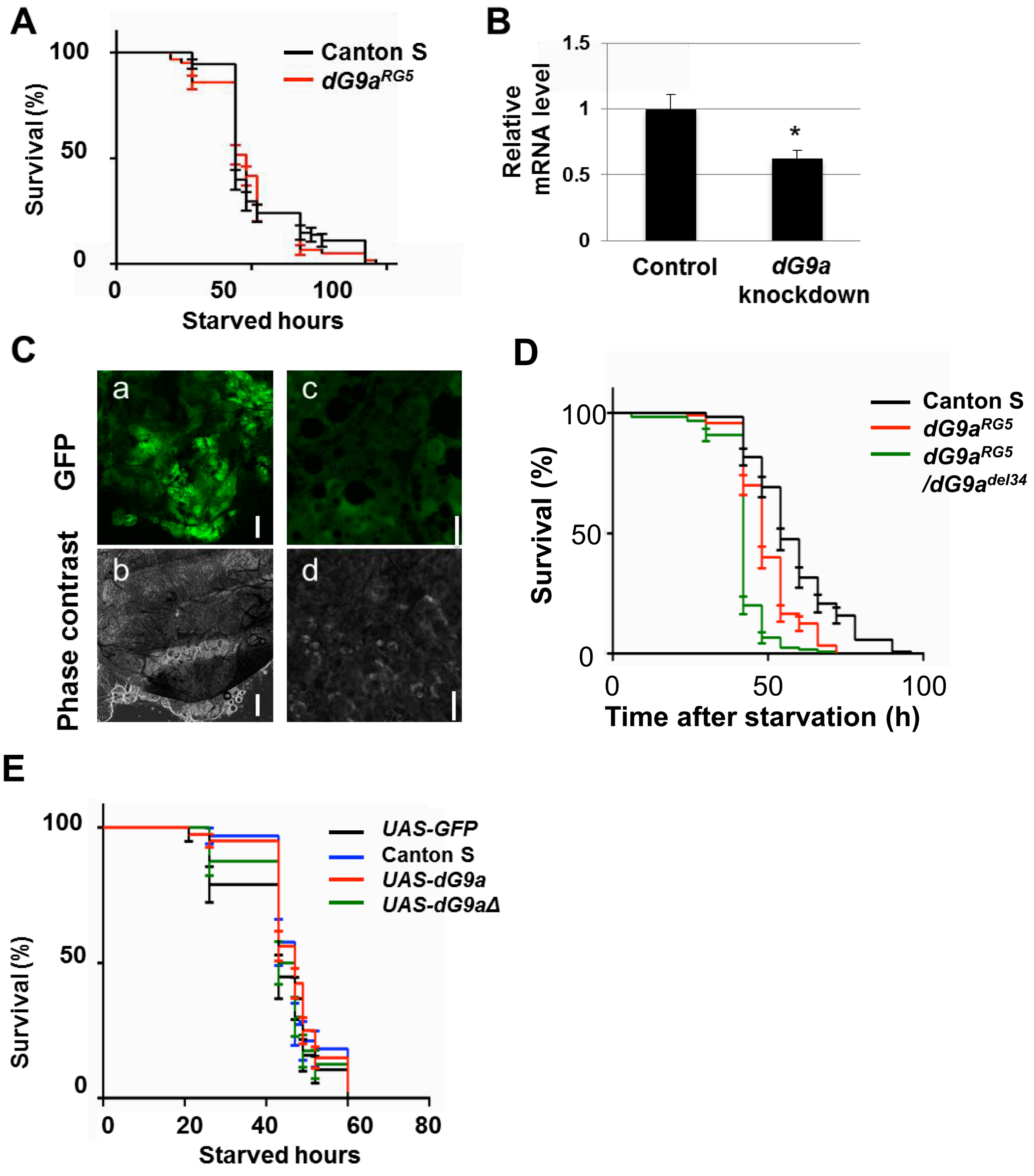


**Figure S1. (A)** The result of viability assay under starvation conditions using larvae of wild type and *dG9a*$^{RG5}$ mutant (n=120 from 6 independent experiments). *P* >0.05. **(B)** Quantification of mRNA level of dG9a in fat-body specifically dG9a knockdown strain (w; FB-GAL4/UAS-FLAG-IR dG9a ;+) and control strain (w; FB-GAL4/+;+). n=3. *P<0.05. **(C)** Immunostaining with anti-GFP antibody in the adult abdominal fat body. The used fly

strain was *P(GAL4)fat; UAS-GFP*. Scale bars: (a,b) 0.9 mm, (c,d) 100µm. **(D)** The result of viability assay under starvation conditions using adult female of Canton S, *dG9a*$^{RG5}$ and *dG9a*$^{RG5}$/ *dG9a*$^{del34}$. n=100 from 5 independent experiments. Error bars represent standard errors (SE). Significant differences were observed between Canton S and *dG9a*$^{RG5}$ (P <0.0001), between Canton S and *dG9a*$^{RG5}$/ *dG9a*$^{del34}$ (P <0.0001) and between *dG9a*$^{RG5}$ and *dG9a*$^{RG5}$/ *dG9a*$^{del34}$ (P <0.0001). *dG9a*$^{RG5}$ lacks the whole open reading frame (ORF) of *dG9a,* however, *dG9a*$^{del34}$ lacks a part of *CG3038* including its transcription start site as well as almost all of the ORF of *dG9a* [21]. *CG3038* is suggested to have glycosyl transferase activity based on the sequence and structural similarity (Flybase:http://flybase.org/). The possible reduction of *CG3038* may result in the further reduction of starvation tolerance in *dG9a*$^{RG5}$/ *dG9a*$^{del34}$ compared with *dG9a*$^{RG5}$ flies. **(E)** The results of viability assay under starvation conditions utilizing male flies of *UAS-GFP* (*UAS-GFP/*+) (n=38 from 2 independent experiments), Canton S (n=33 from 2 independent experiments), *UAS-dG9aΔ* (*UAS-dG9aΔ/*+) (n=40 from 2 independent experiments) and *UAS-dG9a* (*UAS-dG9a/*+) (n=80 from 4 independent experiments). *P* >0.05.

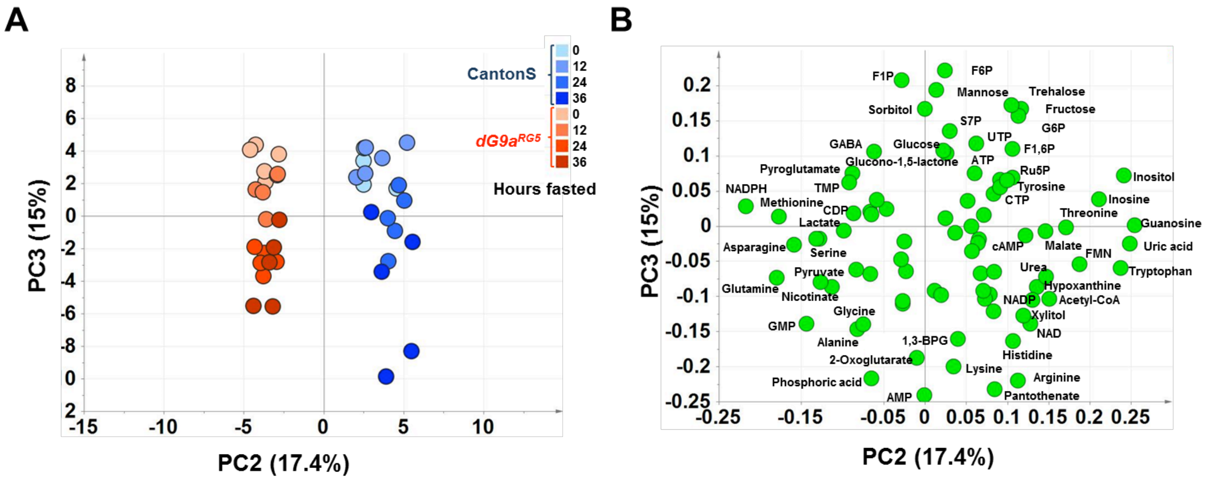


**Figure S2. Metabolic profile of wild type and *dG9a$^{RG5}$* mutant flies under starvation.** PCA score plot (upper panel) constructed by PC2 (17.4%) and PC3 (15%) shows the discriminations in genotypes and in various time points under starvation. This discrimination is explained by the composition of metabolites on loading plot (lower panel). 4-5 replications for each time point.

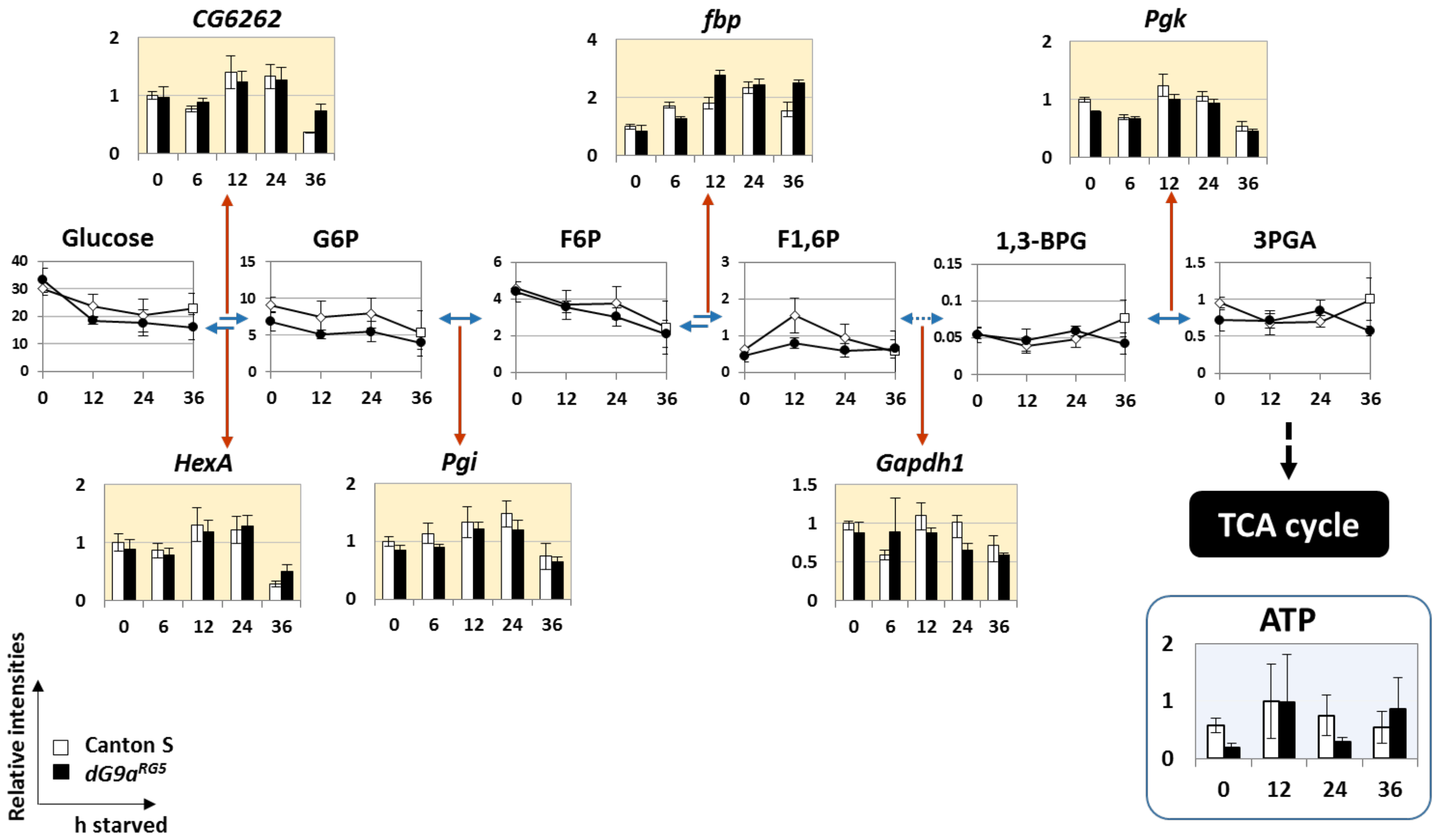


**Figure S3. Glycolysis pathway of wild type and *dG9a*$^{RG5}$ mutant flies during fasting.** The line graphs showing the changes of metabolites. 4-5 replications for each time point. The bar graphs showing the mRNA level of enzymes related to glycolysis. 1,3-BPG: 1,3-Biphosphoglycerate; 3PGA: 3-Phosphoglycerate; F6P: Fructose 6-phosphate; F1,6P: Fructose 1,6-phosphate; G6P: Glucose 6-phosphate

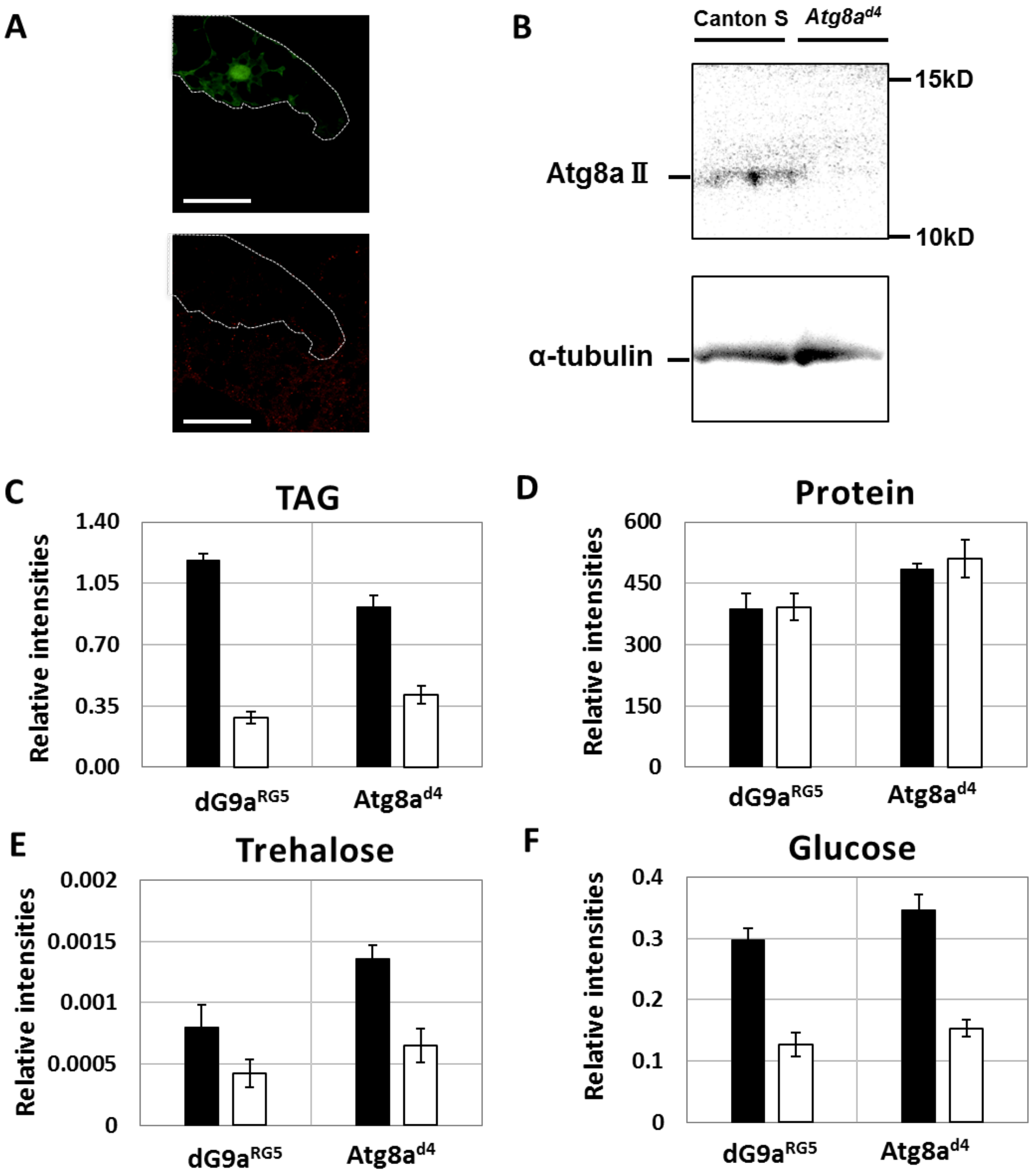


**Figure S4. (A)** Immunostaining of the larval fat body with anti-Atg8a IgG (α-Atg8a) and anti-GFP IgG (α-GFP). Atg8a down-regulated clones marked by GFP were generated in larval fat body. Scale bars: 50μm. **(B)** A western blot analysis of extracts from Canton S and *Atg8a*$^{d4}$. Blots were probed with anti-Atg8a and anti-α-tubulin antibodies. The comparison of TAG, total protein, trehalose and glucose levels between *dG9a*$^{RG5}$ and *Atg8a*$^{d4}$ were shown in **(C), (D), (E)** and **(F)**, respectively. No significant differences were observed between *dG9a*$^{RG5}$ and *Atg8a*$^{d4}$ at 24 h after starvation. Error bars represent SE.

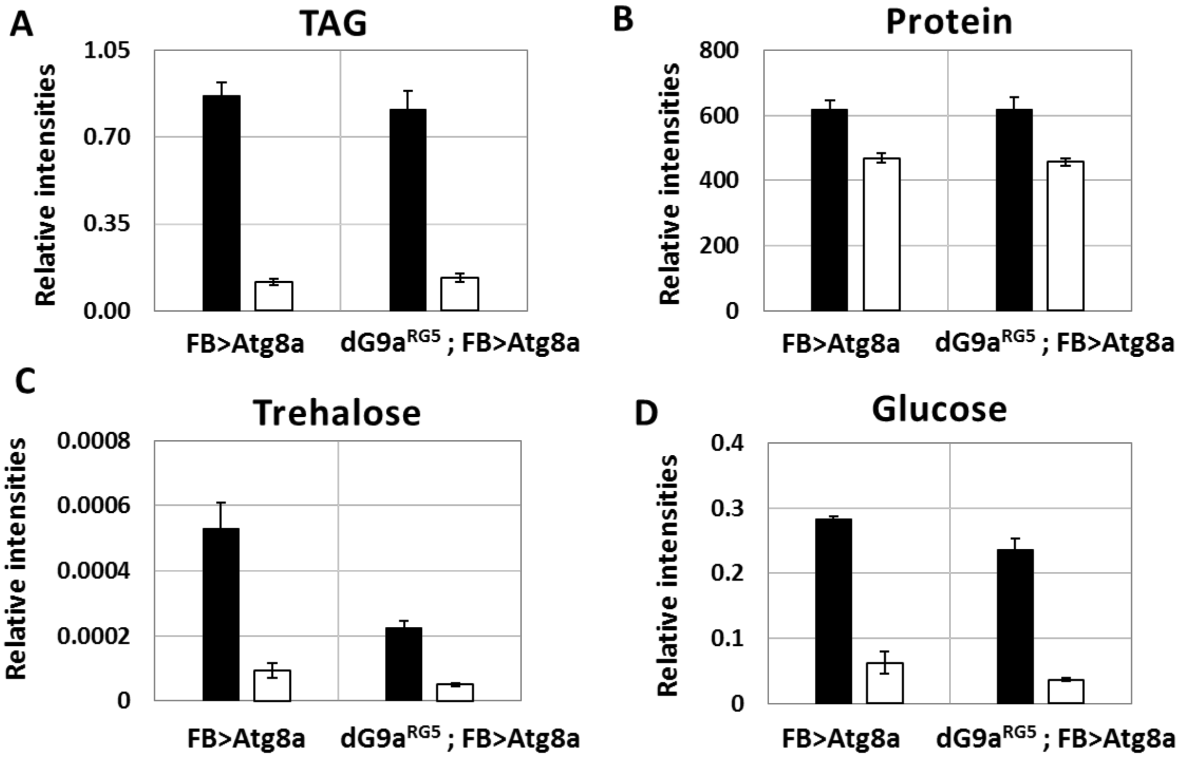


**Figure S5.** The comparison of energy reservoirs between the males of $dG9a^{RG5}$, FB>Atg8a ($dG9a^{RG5}$; $FB\text{-}GAL4/\ Atg8a^{Scer/UAS.P/T.T:Avic/GFP\text{-}EGFP,T:Disc/RFP\text{-}mCherry}$; +) (n=39 from 2 independent experiments) and FB>Atg8a (+; $FB\text{-}GAL4/\ Atg8a^{Scer/UAS.P/T.T:Avic/GFP\text{-}EGFP,T:Disc/RFP\text{-}mCherry}$; +) (n=38 from 2 independent experiments) strains. The level of TAG, total protein, trehalose and glucose were shown in **(A), (B), (C),** and **(D)**, respectively. No significant differences were observed at 24 h after starvation. Error bars represent SE.

**A**

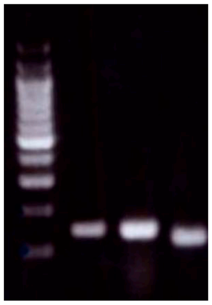

**B**

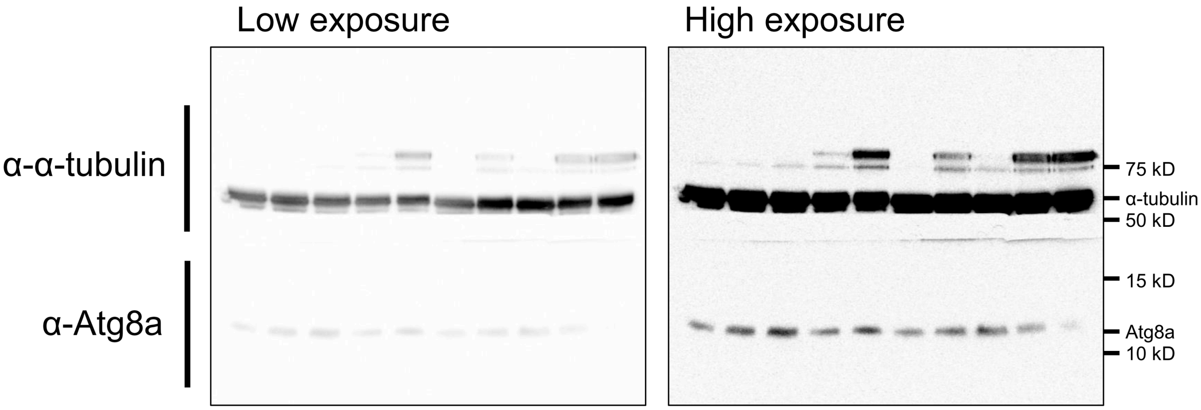


**Figure S6. (A)** The full-length image of Figure 2F. **(B)** The full-length image of Figure 6B.
